# Searchable Encryption Scheme for Personalized Privacy in IoT-Based Big Data

**DOI:** 10.3390/s19051059

**Published:** 2019-03-01

**Authors:** Shuai Li, Miao Li, Haitao Xu, Xianwei Zhou

**Affiliations:** School of Computer and Communication Engineering, University of Science and Technology Beijing, Beijing 100083, China; lis198707@gmail.com (S.L.); lmiao1021@gmail.com (M.L.); xwzhouli@sina.com (X.Z.)

**Keywords:** Internet of Things, big data, searchable encryption, personalized privacy needs, index indistinguishability, trapdoor indistinguishability

## Abstract

The Internet of things (IoT) has become a significant part of our daily life. Composed of millions of intelligent devices, IoT can interconnect people with the physical world. With the development of IoT technology, the amount of data generated by sensors or devices is increasing dramatically. IoT-based big data has become a very active research area. One of the key issues in IoT-based big data is ensuring the utility of data while preserving privacy. In this paper, we deal with the protection of big data privacy in the data storage phase and propose a searchable encryption scheme satisfying personalized privacy needs. Our proposed scheme works for all file types including text, audio, image, video, etc., and meets different privacy needs of different individuals at the expense of high storage cost. We also show that our proposed scheme satisfies index indistinguishability and trapdoor indistinguishability.

## 1. Introduction

Internet of Things (IoT) has become a significant part of our daily life over the past few years. A huge number of sensors or intelligent devices have been integrated together to interconnect people with the physical world, which also generates massive sensing data. Data generated by IoT devices are collected, disseminated, and exchanged among different people, business, and societies. With the development of IoT, the amount of data generated by organizations or individuals is increasing dramatically [1].

Although the massive data generated in the IoT environment is of significant value, exploring and using the extraordinary value of IoT data will increase the risk of privacy breach [2]. To obtain profits, the collection, storage, and reuse of our personal data poses a serious threat to our privacy. Consequently, researchers are faced with the challenge of ensuring the utility of data while preserving privacy. Various techniques have been developed to protect data privacy. Generally, these techniques for data privacy can be grouped based on the stages of big data life cycle, as follows [3].

- Data generation: In the data generation phase, access restriction, and falsifying data techniques are used.
- Data storage: The approaches in the data storage phase are mainly based on encryption techniques.
- Data processing: Anonymization techniques as well as clustering, classification, and association rule mining-based techniques are used in the data processing phase.

In this paper, we will focus on the protection of big data privacy in the data storage phase of the big data life cycle. In the IoT environment, the sensing data generated by various sensors and devices will be collected and uploaded to cloud servers, where cloud servers can provide massive storage and cloud computing services. We know that encryption techniques are used for the protection of big data privacy in the data storage phase. When a large amount of encrypted data is stored in cloud servers, the first consideration is confidentiality of the data, which can be ensured by secure and efficient encryption schemes. However, when the data user wants to retrieve the data containing a specific keyword, the cloud server cannot respond to the data user’s retrieval request, because it cannot decrypt the encrypted data. All these problems can be solved by searchable encryption schemes [4,5], such as searchable symmetric encryption [6], public key encryption with keyword search [7], etc. The searchable encryption scheme mainly includes three entities—data owner, data user, and cloud server. The data owner outsources the encrypted data to the cloud server. The data user queries the encrypted data containing a specific keyword to the cloud server. The cloud server stores and retrieves the encrypted data.

In existing searchable encryption schemes, the data user can access all the data owned by the data owner, which can result in a privacy breach for the data owner. On the one hand, the data owner may be willing to share the data with some specific data users, but not with other data users. On the other hand, the data owner may be willing to share specific data with the data user, but not willing to share other data. Therefore, the data user accesses all the data owned by the data owner, which can result in a privacy breach for the data owner. Furthermore, additional information in the data owned by the data owner can also result in a privacy breach for the data owner. Privacy is subjective, and different people have different privacy needs. For example, the hidden text in a typical Word file includes a lot of sensitive personal information [8]. However, this additional information, which may disclose the privacy of the data owner, is useless for some data users. In data mining, data preprocessing is used to transform raw data into an understandable format [9]. In natural language processing, text feature extraction is used to transform a list of words into a feature set that is usable by a classifier [10]. In speech recognition and image recognition, feature extraction is a key step [11,12]. It means that this additional information may be discarded by the data user in the feature extraction phase. In summary, the data user accessing all the data owned by the data owner will result in a privacy breach for the data owner, but will not improve the utility of the data.

In this paper, we will propose a searchable encryption scheme for personalized privacy protection in IoT-based big data. The main contributions of our proposed scheme are as follows:

- In our proposed scheme, the data owner generates the file features at different levels, and uploads the encrypted file features to the cloud server.
- The proposed scheme makes a trade-off between ensuring the utility of the data and preserving the privacy, and meets the different privacy needs of different individuals.

The rest of this paper is as follows. Section 2 discusses the recent searchable encryption scheme. Section 3 presents necessary notations and definitions. Section 4 formalizes the searchable encryption scheme for meeting the personalized privacy needs in big data and presents main security definition. Section 5 describes the detailed construction of our proposed scheme. Section 6 discusses the security of our proposed scheme. Section 7 performs real time experimental results and makes a comparison of our proposed scheme with the existing schemes. The last section is the conclusion of this paper.

## 2. Related Work

Several different searchable encryption schemes have been proposed to allow the data user to retrieve the encrypted data [4,5]. In this section, we give a simple review on the existing work of the searchable encryption schemes.

In 2000, Song et al. [6] first proposed a searchable encryption scheme based on the symmetric encryption algorithm, which is called searchable symmetric encryption (SSE). However, their scheme has the following limitations: it is not proven to be a secure searchable encryption scheme; the distribution of the underlying plaintexts is vulnerable to statistical attacks; and the search time is linear to the length of the document collection. To overcome these limitations, Goh et al. [13] and Chang and Mitzenmacher [14] deployed a masked index table for SSE and introduced the notion of security for indexes. Curtmola et al. [15] generalized the security definitions of SSE and proposed two SSE schemes which are secure under the new security definitions. The search time of their schemes is linear to the number of documents. Subsequently, several SSE schemes were proposed for improvement. For example, Cash et al. [16] proposed an SSE scheme that supports conjunctive search and general Boolean queries on outsourced symmetrically encrypted data; Salam et al. [17] proposed a privacy-preserving data storage and retrieval system in cloud computing; Li et al. [18] proposed three different SSE schemes that can guard against a coercer by using the deniable encryption idea; Soleimanian et al. [19] proposed an SSE scheme to be publicly verifiable.

Although SSE schemes have high efficiency, they suffer from complicated secret key distribution. To resolve this problem, Boneh et al. [7] introduced a searchable encryption scheme based on public key cryptography, namely public key encryption with keyword search (PEKS). Waters et al. [20] showed that the PEKS schemes based on bilinear map could be applied to build encrypted and searchable auditing logs. However, the bilinear pairing operation is very complicated. Di et al. [21] introduced a PEKS scheme without bilinear pairing. The original PEKS scheme in [7] requires a secure channel to transmit the trapdoors. To overcome this limitation, Baek et al. [22] proposed a new PEKS scheme without requiring a secure channel. Byun et al. [23] introduced the off-line keyword-guessing attack (KGA) and pointed out that the original PEKS scheme in [7] was susceptible to KGA. Rhee et al. [24] proposed the notion of trapdoor indistinguishability and showed that trapdoor indistinguishability is a sufficient condition for preventing outside KGAs. Jeong et al. [25] showed that constructing secure PEKS schemes against inside KGA is impossible under the original PEKS framework in [7]. Xu et al. [26] proposed a PEKS scheme to against inside KGA. More recently, various improved PEKS schemes have been proposed. For example, Liang et al. [27] proposed a searchable attribute-based proxy re-encryption system to achieve privacy-preserving keyword search and encrypted data sharing as well as keyword update; Chen et al. [28] proposed a dual-server PEKS scheme to against inside KGA launched by the malicious server; Yang et al. [29] proposed a semantic key word searchable proxy re-encryption scheme for secure cloud storage using lattice-based cryptographic primitives; Wu et al. [30] designed an efficient and secure searchable encryption protocol using the trapdoor permutation function for cloud-based IoT; Yin et al. [31] proposed a ciphertext-policy attribute-based searchable encryption scheme to achieve keyword-based search and fine-grained access control over encrypted data.

Table 1 shows a simple comparison of some existing searchable encryption schemes. In the design of searchable encryption scheme, privacy is a key concern. However, in all the existing searchable encryption schemes, the data user can access all the data owned by the data owner, which can result in a privacy breach for the data owner.

## 3. Preliminaries

A summary of the notations used in this paper is presented in Table 2.

The set of all binary strings of length *n* is denoted as ${\left\{0,1\right\}}^{n}$, and the set of all finite binary strings is denoted as ${\left\{0,1\right\}}^{*}$.

An index table (or dictionary) denotes the data structure of the form I[key]=value. Given a key, the value matching the key is returned.

A function μ:N→N is negligible if for every positive polynomial p(·) and all sufficiently large λ, $\mu \left(\lambda \right)<\frac{1}{p(\lambda )}$. We similarly write f(λ)=negl(λ) to mean that there exists a negligible function μ(·) such that f(λ)≤μ(λ) for all sufficiently large λ.

The following basic cryptographic primitives can be found in [32].

A symmetric encryption scheme is a tuple E=(Gen,Enc,Dec) of probabilistic, polynomial-time (PPT) algorithms, where Gen takes the security parameter λ as input, and outputs a secret key *k*; Enc takes a key *k* and a message $m\in {\left\{0,1\right\}}^{*}$ as input, and outputs a ciphertext c=Enc(k,m); Dec takes a key *k* and a ciphertext *c* as input, and outputs *m* if c=Enc(k,m).

For any symmetric encryption scheme E=(Gen,Enc,Dec), any adversary A and any value λ for the security parameter, the chosen-plaintext attack (CPA) indistinguishability experiment $SE_{A,E}^{cpa}\left(\lambda \right)$ is defined as:

1. A random key *k* is generated by running Gen(λ).
2. The adversary A is given input λ and oracle access to Enc(k,·), and outputs a pair of messages $m_{0}$, $m_{1}$ of the same length.
3. A random bit b∈{0,1} is chosen, and then a ciphertext $c=Enc(k,m_{b})$ is computed and given to A. *c* is called the challenge ciphertext.
4. The adversary A continues to have oracle access to Enc(k,·), and outputs a bit $b^{\prime }$.
5. The output of the experiment is defined to be 1 if $b^{\prime }=b$, and 0 otherwise. In the case $SE_{A,E}^{cpa}\left(\lambda \right)=1$, we say that A succeeded.

**Definition** **1.** *A symmetric encryption scheme* E=(Gen,Enc,Dec) *is CPA-secure if for all PPT adversaries A there exists a negligible function* negl *such that*

$$\begin{array}{c} Pr\left[SE_{A,E}^{cpa}\left(\lambda \right)=1\right]\leq \frac{1}{2}+\mathrm{negl}\left(\lambda \right), \end{array}$$

*where the probability is taken over the random coins used by A, as well as the random coins used in the CPA indistinguishability experiment.*

For any adversary A and any value λ for the security parameter, the computational Diffie-Hellman (CDH) experiment $CDH_{A,Setup}\left(\lambda \right)$ is defined as:

1. Run Setup(λ) to obtain output (G,q,g), where G is a cyclic group of order *q* (with bit length λ) and *g* is a generator of G.
2. Randomly choose *a*, $b\in Z_{q}$.
3. A is given G, *q*, *g*, $g^{a}$, $g^{b}$ and outputs h∈G.
4. The output of the experiment is defined to be 1 if $h=g^{ab}$, and 0 otherwise.

**Definition** **2.** *The CDH problem is hard relative to* Setup *if for all PPT adversaries A there exists a negligible function* negl *such that*

$$\begin{array}{c} Pr[CDH_{A,Setup}\left(\lambda \right)=1]\leq \mathrm{negl}\left(\lambda \right). \end{array}$$

## 4. System Model

The searchable encryption scheme for personalized privacy protection mainly includes three entities, i.e., the data owner, the data user, and cloud server. The data owner outsources the encrypted file features to the cloud server. The data user queries the encrypted file features containing a specific keyword to the cloud server. The cloud server stores and retrieves the encrypted file features. As the existing searchable encryption schemes, in this paper, the data owner is considered fully trusted. The data user is considered malicious, which means it may attempt to learn more information than it can retrieve. The cloud server is considered honest but curious in the sense that it may try to learn as much information as possible from the stored encrypted data and correctly execute the searchable encryption protocol.

Given *n* files $F_{i}$, 1≤i≤n, and a non-negative integer *l*, let $F_{il}$ denote the file feature of $F_{i}$ at level *l*. Specially, let $F_{i0}=F_{i}$, i.e., the file feature of $F_{i}$ at level 0 is still $F_{i}$.

Let $n_{f}+1$ denote the number of the file feature level (FFL). The data owner wishes to store the file features set $F=\{F_{il}:1\leq i\leq n,0\leq l\leq n_{f}\}$ on the cloud server. The objectives of the data owner are as follows:

- For 1≤i≤n, $0\leq l\leq n_{f}$, the file feature $F_{il}$ are stored on the cloud server such that the confidentiality of $F_{il}$ is preserved.
- The data user queries for a keyword *w* and an FFL *l* to retrieve all authorized file features $F_{il}$ such that $w\in F_{il_{0}}$ for a given $l_{0}$ in a secure and efficient way.

### 4.1. Formal Definition

The searchable encryption scheme for meeting the personalized privacy needs consists of the following algorithms:

- Setup(λ): This algorithm is run by the data owner. It takes the security parameter λ as input, and outputs the global parameter Λ.
- KeyGen(Λ): This algorithm is run by the data owner and the data user, respectively. It takes the global parameter Λ as input, and outputs public/private key pairs $\left(pk_{o},sk_{o}\right)$ and $\left(pk_{u},sk_{u}\right)$ for the data owner and the data user, respectively.
- $Store(F,pk_{u},sk_{o})$: This algorithm is run by the data owner. It takes the file features set F, the data user’s public key $pk_{u}$ and the data owner’s private key $sk_{o}$ as input, and outputs the encrypted file features set $F^{\prime }$ and the encrypted index set $Ind^{\prime }$.
- $Trapdoor(w,l,pk_{o},sk_{u})$: This algorithm is run by the data user. It takes a keyword *w*, an FFL *l*, the data owner’s public key $pk_{o}$, and the data user’s private key $sk_{u}$ as input, and outputs the trapdoor $T_{w,l}$.
- $Search(F^{\prime },Ind^{\prime },T_{w,l})$: This algorithm is performed interactively between the cloud server and the data user. It takes the encrypted file features set $F^{\prime }$, the encrypted index set $Ind^{\prime }$, and the trapdoor $T_{w,l}$ as input, and outputs all authorization file features $F_{il}$ such that $w\in F_{il_{0}}$ for a given $l_{0}$.

### 4.2. Security Definition

The searchable encryption scheme for meeting the personalized privacy needs must satisfy the index indistinguishability and the trapdoor indistinguishability under chosen keyword-FFL pair attack. As per literature [15], we define two challenge-response games $Game_{I}$ and $Game_{T}$ between the adversary A and the challenger C to show the index indistinguishability and the trapdoor indistinguishability under chosen keyword-FFL pair attack, respectively.

The adversary A plays $Game_{I}$ with the challenger C and attempts to distinguish an encrypted index of the given keyword-FFL pair from some encrypted indexes. If A wins $Game_{I}$, then A has obtained some useful information from some encrypted indexes.

  $Game_{I}$:

**Setup:**  Challenger C runs Setup(λ) and KeyGen(Λ) to generate the global parameter Λ and the public/private key pairs $\left(pk_{o},sk_{o}\right)$ and $\left(pk_{u},sk_{u}\right)$ of the data owner and the data user respectively, and sends Λ, $pk_{o}$ and $pk_{u}$ to A.
**Adaptive** **query:** The adversary A makes the following queries to C:
  - The adversary A adaptively selects the keyword-FFL pair (w,l) for the encrypted index query. C responds with $Ind^{\prime }\left[w^{\prime }\right]$.
  - The adversary A adaptively selects the keyword-FFL pair (w,l) for the trapdoor query. C responds with $T_{w,l}$.
**Challenge:**  The adversary A sends two challenged keyword-FFL pairs $\left(w_{0},l_{0}\right)$, $\left(w_{1},l_{1}\right)$ to C. C picks a random number b∈{0,1} and sends the encrypted index $Ind^{\prime }\left[w_{b}^{\prime }\right]$ of the keyword-FFL pair $\left(w_{b},l_{b}\right)$ to A.
**Guess:**  The adversary A outputs $b^{\prime }\in \left\{0,1\right\}$ and wins the game if $b^{\prime }=b$.

**Definition** **3.** *We say the searchable encryption scheme for meeting the personalized privacy needs satisfies the index indistinguishability under chosen keyword-FFL pair attack if for all PPT adversaries A there exists a negligible function* negl *such that*

$$Pr\left[A\;wins\;Game_{I}\right]\leq \frac{1}{2}+\mathrm{negl}\left(\lambda \right).$$

Adversary A plays $Game_{T}$ with challenger C and attempts to distinguish a trapdoor of the given keyword-FFL pair from some trapdoors. If A wins $Game_{T}$, then A has obtained some useful information from some trapdoors.

  $Game_{T}$:

**Setup:**  C runs Setup(λ) and KeyGen(λ) to generate the global parameter Λ and the public/private key pairs $\left(pk_{o},sk_{o}\right)$ and $\left(pk_{u},sk_{u}\right)$ of the data owner and the data user respectively, and sends Λ, $pk_{o}$ and $pk_{u}$ to A.
**Adaptive** **query:** A makes the following queries to C:
  - Adversary A adaptively selects the keyword-FFL pair (w,l) for the encrypted index query. C responds with $Ind^{\prime }\left[w^{\prime }\right]$.
  - Adversary A adaptively selects the keyword-FFL pair (w,l) for the trapdoor query. C responds with $T_{w,l}$.
**Challenge:**  Adversary A sends two challenged keyword-FFL pairs $\left(w_{0},l_{0}\right)$, $\left(w_{1},l_{1}\right)$ to C. C picks a random number b∈{0,1} and sends the trapdoor $T_{w_{b},l_{b}}$ of the keyword-FFL pair $\left(w_{b},l_{b}\right)$ to A.
**Guess:**  Adversary A outputs $b^{\prime }\in \left\{0,1\right\}$ and wins the game if $b^{\prime }=b$.

**Definition** **4.** *We say the searchable encryption scheme for meeting the personalized privacy needs satisfies the trapdoor indistinguishability under chosen keyword-FFL pair attack if for all PPT adversaries A there exists a negligible function* negl *such that*

$$Pr\left[A\;wins\;Game_{T}\right]\leq \frac{1}{2}+\mathrm{negl}\left(\lambda \right).$$

## 5. Proposed Scheme

In this section, we present our proposed searchable encryption scheme for meeting the personalized privacy needs. It consists of the following algorithms.

Setup(λ) is run by the data owner. It takes the security parameter λ as input, and performs the following:

1. Choose a cyclic group G of prime order *q* and a generator *g* of G.
2. Choose a symmetric encryption scheme E=(Gen,Enc,Dec).
3. Choose two collision-resistant hash functions $H_{1}:G\to {\left\{0,1\right\}}^{\lambda }$ and $H_{2}:{\left\{0,1\right\}}^{*}\to {\left\{0,1\right\}}^{\lambda }$.
4. Set the global parameter $\Lambda =(G,q,g,E,H_{1},H_{2})$.

KeyGen(Λ) is run by the data owner and the data user, respectively. It takes the global parameter Λ as input, and performs the following:

1. Randomly select two elements $k_{o}$ and $k_{u}$ in $Z_{q}$ as the private keys of the data owner and the data user, respectively.
2. Compute $g^{k_{o}}$ and $g^{k_{u}}$ in G as the public keys of the data owner and the data user, respectively.

$Store(F,pk_{u},sk_{o})$ is run by the data owner. It takes the file features set F, the data user’s public key $pk_{u}=g^{k_{u}}$ and the data owner’s private key $sk_{o}=k_{o}$ as input, and performs the following:

1. Compute $k_{1}=H_{1}\left({\left(g^{k_{u}}\right)}^{k_{o}}\right)$.
2. For 1≤i≤n, $0\leq l\leq n_{f}$, randomly select $id_{il}\in {\left\{0,1\right\}}^{\lambda }$ as the identifier of $F_{il}$, run algorithm Gen(λ) to generate the encryption key $ek_{il}$ of $F_{il}$, and compute $id_{il}^{\prime }=Enc\left(k_{1},id_{il}\right)$, $ek_{il}^{\prime }=Enc\left(k_{1},ek_{il}\right)$, $F_{il}^{\prime }=Enc\left(ek_{il},F_{il}\right)$.
3. Create the index table $F^{\prime }$ such that $F^{\prime }\left[id_{il}\right]=F_{il}^{\prime }$ for every 1≤i≤n and $0\leq l\leq n_{f}$.
4. Given an FFL $l_{0}$, create the keyword set $W_{l_{0}}$ of the file features set $\left\{F_{il_{0}}:1\leq i\leq n\right\}$.
5. For $w\in W_{l_{0}}$, compute $w^{\prime }=Enc\left(k_{1},H_{2}\left(w\right)\right)$.
6. For $0\leq l\leq n_{f}$, compute $l^{\prime }=Enc\left(k_{1},H_{2}\left(l\right)\right)$.
7. For 1≤i≤n, construct the set $L_{i}$ of the authorized FFL of the file $F_{i}$. In other words, $l\in L_{i}$ implies the date user has authorization to access the file feature $F_{il}$.
8. Create the index table $Ind^{\prime }$ such that $Ind^{\prime }\left[w^{\prime }\right]=\left\{\left(id_{il}^{\prime },ek_{il}^{\prime },l^{\prime }\right):w\in F_{il_{0}},l\in L_{i},1\leq i\leq n\right\}$ for every $w\in W_{l_{0}}$.
9. Send $F^{\prime }$ and $Ind^{\prime }$ to the cloud server.

$Trapdoor(w,l,pk_{o},sk_{u})$ is run by the data user. It takes a keyword *w*, an FFL *l*, the data owner’s public key $pk_{o}=g^{k_{o}}$ and the data user’s private key $sk_{u}=k_{u}$ as input, and performs the following:

1. Compute $k_{2}=H_{1}\left({\left(g^{k_{u}}\right)}^{k_{o}}\right)$.
2. Compute $T_{w,l}=Enc\left(k_{2},H_{2}\left(w\right)\right),Enc\left(k_{2},H_{2}\left(l\right)\right)$.

$Search(F^{\prime },Ind^{\prime },T_{w,l})$ is performed interactively between the cloud server and the data user. It takes the encrypted file features set $F^{\prime }$, the encrypted index set $Ind^{\prime }$ and the trapdoor $T_{w,l}$ as input, and performs the following:

1. The cloud server: Given $T_{w,l}=\left(T_{1},T_{2}\right)$, search $Ind^{\prime }\left[T_{1}\right]$ to obtain the set $S=\{\left(s_{1},s_{2},s_{3}\right)\in Ind^{\prime }\left[T_{1}\right]:s_{3}=T_{2}\}$ and send S to the data user.
2. The data user: Given S, create two index tables $S_{1}$ and $S_{2}$ such that $S_{1}\left[r_{s}\right]=Dec\left(k_{2},s_{1}\right)$, $S_{2}\left[r_{s}\right]=Dec\left(k_{2},s_{2}\right)$ for every $s=(s_{1},s_{2},s_{3})\in S$, where $k_{2}=H_{1}\left({\left(g^{k_{u}}\right)}^{k_{o}}\right)$ and $r_{s}$ (s∈S) are randomly selected in ${\left\{0,1\right\}}^{\lambda }$. Send $S_{1}$ to the cloud server and store $S_{2}$.
3. The cloud server: Given $S_{1}$, create the index table R such that $R\left[r_{s}\right]=F^{\prime }\left[S_{1}\left[r_{s}\right]\right]$ for every key $r_{s}$ in $S_{1}$ and send R to the data user.
4. The data user: Given $S_{2}$ and R, compute $Dec(S_{2}\left[r_{s}\right],R\left[r_{s}\right])$ for every key $r_{s}$ in $S_{2}$.

**Remark** **1.** *Please note that*$k_{1}=H_{1}\left({\left(g^{k_{u}}\right)}^{k_{o}}\right)=H_{1}\left({\left(g^{k_{u}}\right)}^{k_{o}}\right)=k_{2}$*, then*$T_{1}=w^{\prime }$, $T_{2}=l^{\prime }$*. Thus,*$s_{1}=id_{il}^{\prime }$*,*$s_{2}=ek_{il}^{\prime }$*,*$S_{1}\left[r_{s}\right]=id_{il}$*,*$S_{2}\left[r_{s}\right]=ek_{il}$*,*$R\left[r_{s}\right]=F^{\prime }\left[S_{1}\left[r_{s}\right]\right]=F_{il}^{\prime }$*for every*$s=(s_{1},s_{2},s_{3})\in S$*, where*$w\in F_{il_{0}}$*,*$l\in L_{i}$*,*1≤i≤n*. Therefore, our proposed scheme is correct.*

Given an FFL $l_{0}$, creating the keyword set $W_{l_{0}}$ of the file features subset $\left\{F_{il_{0}}:1\leq i\leq n\right\}$ means that $F_{il_{0}}$, 1≤i≤n must be text. Thus, our proposed scheme works for all file types including text, audio, image, video, etc. as long as there exists an FFL $l_{0}$ such that the file feature of the file at $l_{0}$ is text.

If the authorized FFL set of the ordinal file is only created by the data owner, then the data user cannot access to the unauthorized file features, thus our proposed scheme meets the different privacy needs of different individuals.

Our proposed scheme can be extended to the multi-user scenario. Let $n_{o}$ and $n_{u}$ be the number of the data owners and the data users, respectively. In the multi-user scenario, the public/private key pairs are first generated for every data owner and the data user; the file features stored on the cloud server is an $n_{o}$-ary vector, where the *i*-th element is the encrypted file features set of the *i*-th data owner; the index stored on the cloud server is an $n_{o}\times n_{u}$ matrix, where the *i*-th row and *j*-th column element is the encrypted index set that the *i*-th data owner created for the *j*-th data user.

It is obvious that our proposed scheme needs increasing storage space when $n_{f}$ is getting bigger. In particular, our proposed scheme has similar storage space to the existing searchable encryption schemes when $n_{f}=0$.

## 6. Security Analysis

In this section, we show that our proposed scheme satisfies the index indistinguishability and the trapdoor indistinguishability under chosen keyword-FFL pair attack.

**Theorem** **1.** *If* E=(Gen,Enc,Dec) *is CPA-Secure and the CDH problem is hard relative to* Setup *, then our proposed scheme satisfies the index indistinguishability under chosen keyword-FFL pair attack.*

**Proof.**  If there exists a PPT, and adversary A wins $Game_{I}$, then there exists a simulator *B* such that $SE_{B,E}^{cpa}\left(\lambda \right)=1$ or $CDH_{B,Setup}^{cpa}\left(\lambda \right)=1$.

In the setup phase, C runs Setup(λ) and KeyGen(Λ) to generate the global parameter $\Lambda =(G,q,g,E,H_{1},H_{2})$, and the public/private key pairs $\left(pk_{o}=g^{k_{o}},sk_{o}=k_{o}\right)$ and $\left(pk_{u}=g^{k_{u}},sk_{u}=k_{u}\right)$ of the data owner and the data user respectively. Then, C sends Λ, $pk_{o}=g^{k_{o}}$ and $pk_{u}=g^{k_{u}}$ to A.

In the adaptive query phase, assume A makes $n_{q}-1$ queries to C adaptively. The *q*-th query can be:

- A adaptively selects the keyword-FFL pair $\left(w_{q},l_{q}\right)$ for the encrypted index query. C responds with $Ind^{\prime }\left[w_{q}^{\prime }\right]=\left\{\left(id_{il_{q}}^{\prime },ek_{il_{q}}^{\prime },l_{q}^{\prime }\right):w_{q}\in D_{i},l_{q}\in L_{i},1\leq i\leq n\right\}$, where $L_{i}$ is the authorized FFL set of $F_{i}$, $id_{il_{q}}^{\prime }=Enc\left(k_{1},id_{il_{q}}\right)$, $ek_{il_{q}}^{\prime }=Enc\left(k_{1},ek_{il_{q}}\right)$, $l_{q}^{\prime }=Enc\left(k_{1},H_{2}\left(l_{q}\right)\right)$, $k_{1}=H_{1}\left({\left(g^{k_{o}}\right)}^{k_{u}}\right)$.
- A adaptively selects the keyword-FFL pair $\left(w_{q},l_{q}\right)$ for the trapdoor query. C responds with $T_{w_{q},l_{q}}=(Enc\left(k_{2},H_{2}\left(w_{q}\right)\right),Enc\left(k_{2},H_{2}\left(l_{q}\right)\right)$, where $k_{2}=H_{1}\left({\left(g^{k_{u}}\right)}^{k_{o}}\right)$.

In the challenge phase, A sends two challenged keyword-FFL pairs $\left(w_{0},l_{0}\right)$, $\left(w_{1},l_{1}\right)$ to C. C picks a random number b∈{0,1} and sends the encrypted index $Ind^{\prime }\left[w_{b}\right]=\left\{\left(id_{il_{b}}^{\prime },ek_{il_{b}}^{\prime },l_{b}\right):w_{b}\in D_{i},l_{b}\in L_{i},1\leq i\leq n\right\}$ of the keyword-FFL pair $\left(w_{b},l_{b}\right)$ to A, where $id_{il_{b}}^{\prime }=Enc\left(k_{1},id_{il_{b}}\right)$, $ek_{il_{b}}^{\prime }=Enc\left(k_{1},ek_{il_{b}}\right)$, $l_{b}^{\prime }=Enc\left(k_{1},H_{2}\left(l_{b}\right)\right)$ and $k_{2}=H_{1}\left({\left(g^{k_{1}}\right)}^{k_{u}}\right)$.

In the guess phase, A outputs its guess $b_{1}\in \left\{0,1\right\}$ indicating whether the challenge $Ind^{\prime }\left[w_{b}\right]$ is the encrypted index of $\left(w_{0},l_{0}\right)$ or $\left(w_{1},l_{1}\right)$.

From the perspective of A, $id_{il_{q}}^{\prime }=Enc\left(k_{1},id_{il_{q}}\right)$ and $ek_{il_{q}}^{\prime }=Enc\left(k_{1},ek_{il_{q}}\right)$ are random values in ${\left\{0,1\right\}}^{\lambda }$ for every 1≤i≤n and $2\leq q\leq n_{q}$. Please note that $k_{1}=H_{1}\left({\left(g^{k_{u}}\right)}^{k_{o}}\right)=H_{1}\left({\left(g^{k_{o}}\right)}^{k_{u}}\right)=k_{2}$. Then the information obtained by the adversary A in $Game_{I}$ was the same as the information obtained by a simulator *B* in the CPA indistinguishability experiment $SE_{A,E}^{cpa}\left(\lambda \right)$ and in the CDH experiment $CDH_{A,Setup}\left(\lambda \right)$. Thus, if A wins $Game_{I}$ then $SE_{B,E}^{cpa}\left(\lambda \right)=1$ or $CDH_{B,Setup}\left(\lambda \right)=1$, i.e.,

$$\begin{array}{ccc} Pr[A\;\mathrm{wins}\;Game_{I}] & \leq & SE_{B,E}^{cpa}\left(\lambda \right)+CDH_{B,setup}\left(\lambda \right)\\ & \leq & \frac{1}{2}+\mathrm{negl}\left(\lambda \right). \end{array}$$

Therefore, our proposed scheme satisfies the index indistinguishability under chosen keyword-FFL pair attack if E=(Gen,Enc,Dec) is CPA-Secure and the CDH problem is hard relative to Setup. □

Similarly, we can prove the following theorem:

**Theorem** **2.** *If*E=(Gen,Enc,Dec)*is CPA-Secure and the CDH problem is hard relative to*Setup*, then our proposed scheme satisfies the trapdoor indistinguishability under chosen keyword-FFL pair attack.*

## 7. Performance Analysis

As shown in Table 3, we present a comprehensive comparison of the computation cost between our proposed scheme and some existing searchable encryption schemes. The notations used in Table 3 are as follows:

1. $T_{bp}$: Time cost for a bilinear pairing.
2. $T_{h}$: Time cost for a hash function.
3. $T_{exp}$: Time cost for an exponentiation operation in G.
4. $T_{mul}$: Time cost for a multiplication operation in G.
5. $T_{enc}$: Time cost for an encryption process of E.
6. $T_{dec}$: Time cost for a decryption process of E.

To meet the basic security level for comparison, SHA-256 and AES-256 is selected as the collision-resistant hash function and the symmetric encryption scheme, respectively. The cyclic group G of order *q* is generated by a point on an elliptic curve $E(F_{p})$, where *q* and *p* are the 256-bits and 521-bits prime numbers, respectively. To evaluate the efficiency of the five schemes, we perform our experiments on a computer with 2.4 GHz Intel Core i7 and 8 GB RAM.

**Figure 1:**
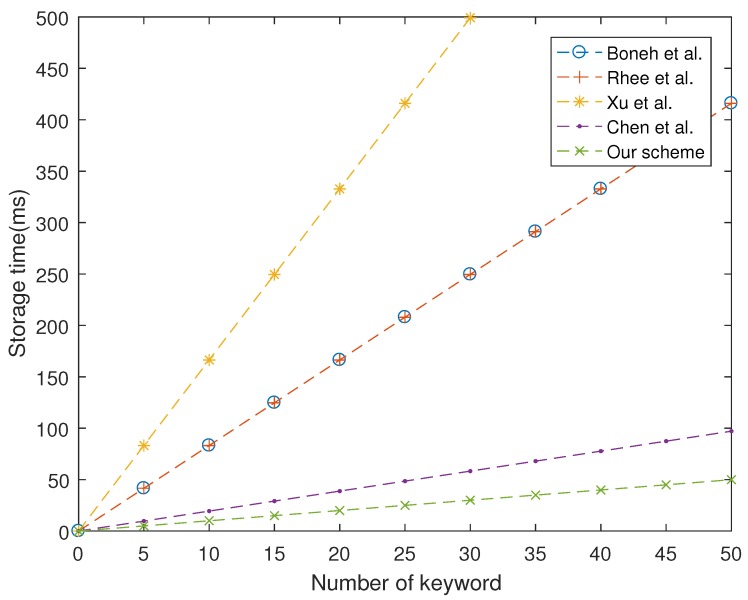
Computation cost at storage phase.

**Figure 2:**
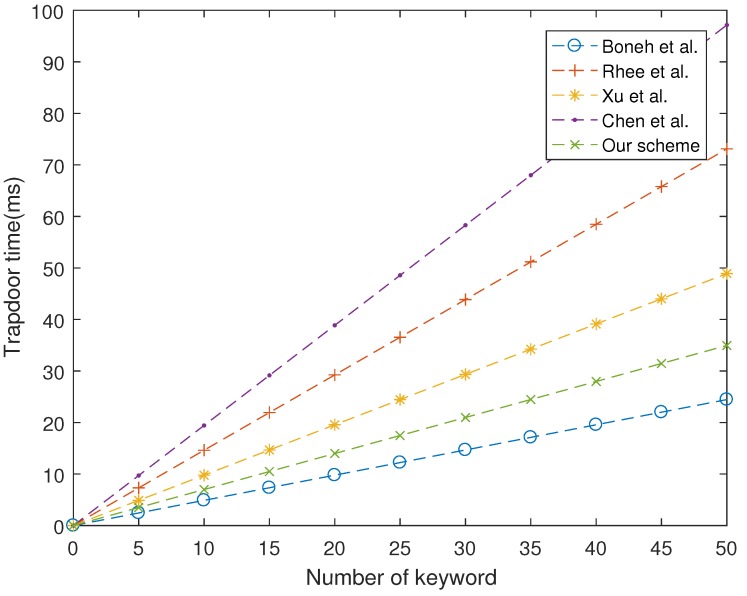
Computation cost at trapdoor phase.

**Figure 3:**
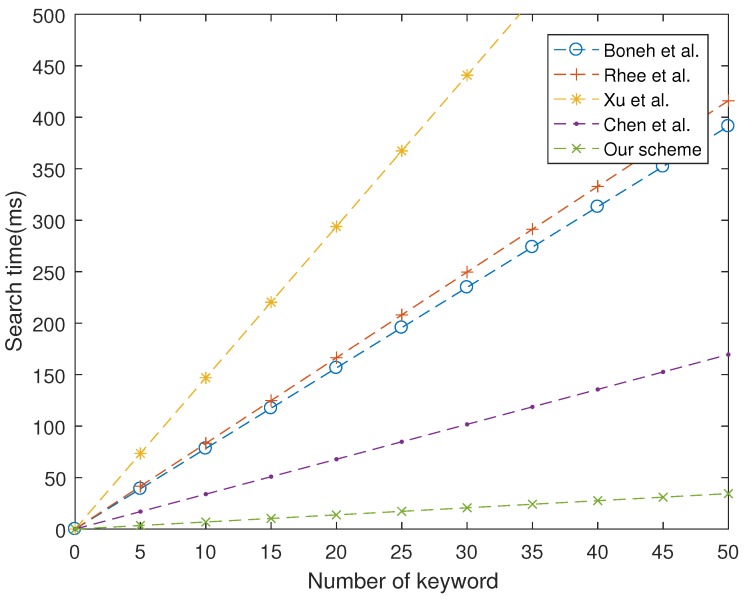
Computation cost at search phase.

As shown in Figure 1, Figure 2 and Figure 3, our proposed scheme is the most efficient in storage phase and search phase. In trapdoor phase, our proposed scheme has a higher computational cost than that of Boneh et al. [7], although it is still lower than other schemes. In summary, the performance of our proposed scheme is more efficient than four schemes studied in [7,24,26,28].

## 8. Conclusions

In this paper, we have proposed a searchable encryption scheme for meeting personalized privacy needs. Our proposed scheme mainly includes three entities, i.e., the data owner, the data user, and cloud server. The data owner outsources the encrypted file features to the cloud server. The data user queries the encrypted file features containing a specific keyword to the cloud server. The cloud server stores and retrieves the encrypted file features. Compared with the existing searchable encryption schemes, our proposed scheme works for all file types including text, audio, image, video, etc., and meets different privacy needs of different individuals at the expense of high storage cost. We also show that our proposed scheme satisfies index indistinguishability and trapdoor indistinguishability under chosen keyword-FFL pair attack. In other words, our proposed scheme is secure against inside KGA. Performance analysis shows that our proposed scheme is efficient in storage phase, trapdoor phase, and search phase.

Considering the decreasing costs of storage, storage cost is not a problem if $n_{f}+1$, i.e., the number of the FFL is small in our proposed scheme. However, storage cost is still a problem if $n_{f}$ is too large in our proposed scheme. Thus, choosing an appropriate $n_{f}$ is an important work in the future.

## Figures and Tables

**Table 1:** A comparison of some existing searchable encryption schemes.

Type	Limitation	Characteristic	Literature
SSE	need key distribution	masked index	[13,14]
		boolean queries	[16]
		against the coercer	[18]
		publicly verifiable	[19]
PEKS	lower search efficiency	without bilinear pairing	[21]
		without secure channel	[22]
		keyword update	[27]
		against inside KGA	[28]
		synonym keyword search	[29]
		fine-grained access control	[31]

**Table 2:** Summary of notations.

Notation	Description
λ	The security parameter
G	A cyclic group of order *q*
*g*	A generator of G
negl(λ)	A negligible function with respect to λ
G	A cyclic group of order *q*
*g*	A generator of G
$\left(pk_{o},sk_{o}\right)$	The public/private key pairs for the data owner
$\left(pk_{u},sk_{u}\right)$	The public/private key pairs for the data user
*n*	The number of the file of the data owner
$F_{i}$	The *i*-th file of the data owner (1≤i≤n)
$n_{f}+1$	The number of the file feature level
*l*	A file feature level ($0\leq l\leq n_{f}$)
$L_{i}$	The set of the authorized file feature level of $F_{i}$
$F_{il}$	The file feature of $F_{i}$ at level *l*
F	The file features set $\left\{F_{il}:1\leq i\leq n,0\leq l\leq n_{f}\right\}$
$F^{\prime }$	The encrypted file features set
$W_{l_{0}}$	The keyword set of the file features set $\left\{F_{il_{0}}:1\leq i\leq n\right\}$
*w*	A keyword in $W_{l_{0}}$
Ind	The index set
$Ind^{\prime }$	The encrypted index set
$T_{w,l}$	The trapdoor with respect to *w* and *l*

**Table 3:** Computation cost: a comprehensive comparison.

Scheme	Computation		
	Storage Phase	Trapdoor Phase	Search Phase
Boneh et al. [7]	$T_{bp}+2T_{h}+2T_{exp}$	$T_{h}+T_{exp}$	$T_{bp}+T_{h}$
Rhee et al. [24]	$T_{bp}+2T_{h}+2T_{exp}$	$2T_{h}+3T_{exp}$	$T_{bp}+2T_{h}+2T_{exp}+T_{mul}$
Xu et al. [26]	$2T_{bp}+4T_{h}+4T_{exp}$	$2T_{h}+2T_{exp}$	$2T_{bp}+2T_{h}$
Chen et al. [28]	$T_{h}+4T_{exp}+2T_{mul}$	$T_{h}+4T_{exp}+2T_{mul}$	$7T_{exp}+3T_{mul}$
Our scheme	$T_{exp}+3T_{h}+5T_{enc}$	$T_{exp}+3T_{h}+2T_{enc}$	$T_{exp}+T_{h}+2T_{dec}$

## Abbreviations

The following abbreviations are used in this manuscript:

IOT	Internet of Things
SSE	searchable symmetric encryption
PKES	public key encryption with keyword search
KGA	keyword guessing attac
FFL	The file feature level
