# Erratum: Li, S.; Miao, L.; Xu, H.; Zhou, X. Searchable Encryption Scheme for Personalized Privacy in IoT-Based Big Data. *Sensors* 2019, *19*, 1059

**DOI:** 10.3390/s19102327

**Published:** 2019-05-20

**Authors:** Sensors Editorial Office

**Affiliations:** MDPI AG St. Alban-Anlage 66, 4052 Basel, Switzerland; sensors@mdpi.com

The *Sensors* Editorial Office wishes to report the following erratum to this paper [1]. In the paper, the funding section states:

“This work is supported by the National Natural Science Foundation of China under Grant (No. U1603116, No. 61701020).”

But an additional funding program must be added:

“This work is supported by the National Key R&D Program of China (No. 2018YFB1003905) and the National Natural Science Foundation of China under Grant (No. U1603116, No. 61701020).”

The error was introduced during production. We apologize for any inconvenience caused to the readers by this mistake. The manuscript will be updated and the original will remain online on the article website.
